# Causality, mediation and time: a dynamic viewpoint

**DOI:** 10.1111/j.1467-985X.2011.01030.x

**Published:** 2012-10

**Authors:** Odd O Aalen, Kjetil Røysland, Jon Michael Gran, Bruno Ledergerber

**Affiliations:** University of OsloNorway; University Hospital ZurichSwitzerland

**Keywords:** Causal inference, Dynamic path analysis, Granger causality, Local independence, Mediation

## Abstract

**Summary.** Time dynamics are often ignored in causal modelling. Clearly, causality must operate in time and we show how this corresponds to a mechanistic, or system, understanding of causality. The established counterfactual definitions of direct and indirect effects depend on an ability to manipulate the mediator which may not hold in practice, and we argue that a mechanistic view may be better. Graphical representations based on local independence graphs and dynamic path analysis are used to facilitate communication as well as providing an overview of the dynamic relations ‘at a glance’. The relationship between causality as understood in a mechanistic and in an interventionist sense is discussed. An example using data from the Swiss HIV Cohort Study is presented.

## 1. Introduction

In recent years causality has become a major issue in statistics. Whereas, previously, statisticians tended to be silent on the issue of causality, we experience today a surge of interest and a feeling that statistical analysis should confront causal issues in a much more active way than has been common previously. Pearl (2000, 2009) has crystallized a number of issues on statistics and causality, and has been a major factor in the rise of causal thinking in statistics. He holds strong views on the previous avoidance of causality in statistical science, stating that

‘… this position of caution and avoidance has paralysed many fields that look to statistics for guidance, especially economics and social science’

(Pearl (2000), page 341).

The term ‘causal inference’ has come to denote certain special ways to approach the causal aspects of statistical analysis. However, there is no ‘magic bullet’ for causality in statistics, and one has to open up for many different ways of viewing this important and difficult issue.

As regards causality we may assume (at least) two different points of view. The dominant view in statistics puts the concept of an intervention at the forefront (Pearl, 2009.) The causal effect of the intervention is judged by comparing the evolution of the system when the intervention is and when it is not present. This is often formulated in a counterfactual way, namely that we can consider the same given system both with and without the intervention, all else being equal, but there are also non-counterfactual formulations of interventionist causality (Dawid, 2000). This type of thinking is very popular in the medical field, which after all is dominated by attempts to intervene to achieve good medical effects.

The other point of view focuses on mechanisms; one wishes to understand how the effects come about (Machamer *et al.*, 2000). No doubt, the great success of natural science is due to the elucidation of the inner workings of nature. A classical example is Newton’s explanation of the tides being due to the gravitational effect of the moon on the waters on the Earth. This may be viewed as a causal relation although one can hardly imagine intervening in this system. Another example is global warming where mechanistic understanding in terms of physical principles tells us that carbon dioxide emission should be a major causal factor. No large-scale intervention has so far been made to document this, although the hope is that in the future one may intervene to reduce carbon dioxide emission and thus alleviate the warming. There are many such situations where we can understand mechanisms, but where intervention has not been realistic or practical, and in medicine often not ethical. Clearly, the term mechanism here should not be taken literally, as a machine with many buttons and levers that you can twist and turn; rather it denotes the inner workings of a system which may be just partly understood.

According to some researchers, the concept of mechanism also includes a productive aspect: an understanding of how things come about or why they happen. For instance, Machamer *et al.* (2000), page 3, defined mechanisms in the following way:

‘Mechanisms are entities and activities organised such that they are productive of regular changes from start or set-up to finish or termination of conditions’.

The interventionist and mechanistic viewpoints are by no means entirely separate. When studying, say, biological mechanisms scientists carry out large numbers of experiments where they intervene on bits and parts of the system, for instance by adding a substance or by knocking out a gene. Still, the effect of many large-scale interventions, e.g. of a medication introduced into the human body, cannot be decided in the laboratory. However, a mechanistic understanding can suggest that a certain medication ought to work and propose it for study. It is clear that the final aim of the biomedical research effort to elucidate mechanisms is in the end to intervene and either to prevent or cure disease. But there is a long road leading to this end, and along the way elaboration of mechanisms is one major tool.

It is also important to note that an interventionist understanding of causality may be acquired without necessarily understanding mechanisms. Obviously humans have always had a large amount of causal knowledge to guide our daily life, and it is only quite recently that natural science has uncovered the underlying mechanisms corresponding to much of this causal knowledge (for example we have always known that certain mushrooms are poisonous, but today the mechanisms behind this will in many cases have been discovered). Also today the basis of much medical practice is based on statistical results from clinical trials and the underlying mechanisms are at most only partially understood (Aalen and Frigessi, 2007; VanderWeele, 2009).

In a sense, mechanisms represent the structure of the world (Salmon, 1984), and the aim of human intervention is to gain understanding of this structure to exploit it for some purpose. The mechanisms in the structure of the world are present whether humans are there to intervene or not, and hence seem to be the more fundamental aspect.

In medical studies, the prototype of a causal question seems to be whether treatment A or treatment B should be administered to future patients to optimize a given (expected) outcome *Y*. In this case it is relatively clear why we are asking a causal question: we want to optimize future actions, namely the administration of a treatment to improve patients’ health. It is commonly accepted (and can be proved in a variety of causal frameworks) that if we conduct a randomized controlled trial comparing these two treatments and collect data on the outcome *Y* we will be able to find out which if any of the two treatments is better, and to choose our future actions accordingly.

Much of the causal literature basically deals with this kind of causal question (or some more subtle versions such as sequential treatments or complier causal effects) and addresses two issues.

(a) If a randomized controlled trial cannot be carried out (e.g. for ethical or financial reasons) under what assumptions can we still consistently estimate this causal effect from observational data?
(b) What methods will give us consistent, efficient and robust estimates of this causal effect (e.g. adjustment for confounding, inverse probability weighting, propensity scores and *G*-estimation)?

These are important questions, but they assume that there is already a very specific causal hypothesis to be investigated (which treatment is better).

Cox (2000) pointed out that if the only evidence we had was that, say, treatment A performed better in a randomized trial than treatment B, without a clue why this is so, most scientists would not be comfortable with recommending treatment A. In fact, this seems like a black box approach that hardly promotes causal understanding. Breslow (2000) made the following interesting statement: ‘Counterfactual causality with its paradigm, randomization, is the ultimate black box,…’. Obviously, in practice there will be a long history of developing, experimenting with and trying out different substances that lead to the treatments, which would have given sufficient reason to want to test them in a controlled trial. It is this kind of exploration that forms the basis of causal understanding, whereas the randomized trial is ‘just’ the formal confirmation.

There should be more attention in statistics to causal exploration, as opposed to confirmation, i.e. with finding out why a certain treatment might be better than another. We might want to try to predict the effect of external changes or shocks to a system in which humans cannot (or only to a very limited extent) intervene. For this task of exploration it is necessary to consider the data-generating processes or mechanisms in as much detail as possible and especially to take their development over time into account. In particular it will be important to distinguish between direct and indirect effects. Statistics as a field has been mainly concerned with carrying out formal evaluations to judge whether we can ‘prove’ a treatment effect, due to the major role of statistics in clinical trials and epidemiological effect studies. This has been to the detriment of actually understanding what goes on inside the system, as stated clearly by Heckman (2008). Hafeman and Schwartz (2009) called for ‘Opening the Black Box’. This is long overdue. Cox (1992) made a strong case for mechanistic understanding as part of causality. An excellent philosophical discussion of causality and the relation to mechanisms is given in Psillos (2002).

We would like to point out that the meaning of a mechanistic understanding depends on the particular scientific setting. In natural science extensive laboratory experiments are carried out and the scientific meaning of the mechanisms can be explored in great detail. In social science and psychology one also thinks in terms of mechanisms, but these mechanisms can to a much lesser extent be subjected to laboratory experiments and their meaning is less clear. It is particularly in the latter area that statistical analysis had been used as a tool for understanding mechanisms as illustrated for instance in Baron and Kenny (1986). However, the promise for statistics to elucidate mechanisms of risk factors and treatment for somatic disease should be greater, due to extensive basic biological knowledge that can be combined with the statistical results.

The aims of this paper are as follows:

(a) to show the importance of a time-based process point of view when studying causality;
(b) to discuss critically the counterfactual definition of mediation direct and indirect effects;
(c) to argue for a more detailed analysis of causal data with the aim of achieving mechanistic understanding;
(d) to show how the concepts of local independence and dynamic path analysis are useful in this context;
(e) to analyse a set of data from the Swiss HIV Cohort Study;
(f) furthermore, in spite of our statistical background we make an attempt to relate the ideas and concepts to real life examples, mainly from medicine. The authors, collectively, have much experience in this area, in particular as regards infection with human immunodeficiency virus (HIV).

We shall start by discussing important concepts before entering the more mathematical issues.

## 2. Modelling causality

### 2.1. Causal directed acyclic graphs and counterfactual worlds

**Figure 1:**
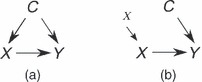
Causal diagram illustrating the influence from an exposure *X* on an outcome *Y*, with the possible influence of a confounder *C*: intervening on *X* to give it a specific value breaks the influence of the confounder

The concept of intervention is important for causality. When focusing on this concept in causal studies, it simplifies the matter considerably if the intervention can be seen as having a simple effect. This idea has been most clearly formulated by Pearl (2000, 2009), who introduced the ‘do’ operator to separate intervention from conditioning and combined this with causal Bayesian networks. Bayesian networks, represented by directed acyclic graphs (DAGs), describe conditional independence relationships between variables and calculations of probabilities can be made in such a network by employing a Markov condition with respect to the parents of a node. A simple illustration is given in the causal diagram of Fig. 1, where an exposure *X* influences an outcome *Y* with a confounder *C*. Fig. 1(a) illustrates the causal dependences without intervention. The idea is that intervention breaks the influence of the confounder so that one can make a true causal assessment of the influence of *X* on *Y*, and this is expressed by Pearl’s do operator. As illustrated in Fig. 1(b) the intervention has the effect of removing the arrow pointing to node *X*, but otherwise no change takes place in the system or the conditional probabilities. This is what Pearl denotes as the surgery of intervention.

Pearl presents his surgery as a generally applicable procedure given an appropriate causal diagram: intervention implies that a node in the corresponding causal diagram is set to a specific value and then deleting all incoming links to this node; otherwise no changes are made in the diagram and the Markov assumption is preserved in the new graph. This simple idea is surprisingly fruitful in assessing the influence of interventions in much more complex situations than that envisaged in Fig. 1 and leads to a nice and general mathematical theory. In fact, Pearl (2000, 2009) defined a causal Bayesian network, or causal DAG, as being a network where the effect of any intervention can be defined by such a surgery procedure. This assumes that intervention is a primitive (undefined) concept, which then defines causality. This raises the issue of what intervention really is, and whether it is necessarily related to human action or requires free will. Intervention necessarily means an impulse that comes from outside the system considered, and it may not be clear in which sense such an ‘outside’ exists; for some interesting philosophical discussion, see Woodward (2008). Undoubtedly, Pearl’s do operator is an important advance, allowing elegant mathematical analysis of complex situations as demonstrated in Pearl (2000, 2009); for related material see also Spirtes *et al.* (2000).

Pearl’s do operator seems to presume a rather strict and definitive concept of intervention. In a medical setting interventions will often have a more vague and uncertain character. For instance, a person may decide to stop smoking (the intervention), but from general experience it is quite possible that he may relapse and start smoking again. Hence, the intervention induces a change of state that will influence future observations and processes and this does not seem to fit well with Pearl’s intervention concept. The prominent philosopher Nancy Cartwright in Cartwright (2007) also asserted that intervention in practice will usually be a much more complex affair than what is indicated by Pearl’s surgery. She states in her book about interventions:

‘For policy and evaluation we generally want to know what would happen were the policy really set in place. And whatever we know about how it might be put in place, the one thing we can usually be sure of is that it will not be by a precise incision of the kind Pearl assumes. … We implement a policy to ensure that it will obtain–and the policy may affect a host of changes in other variables in the system, some envisaged and some not.’

Similar arguments are presented by Heckman (2005). Pearl (2009), section 11.4.6, quotes Cartwright extensively and argues against her, stating for example that

‘surgery is a symbolic operation which makes no claims about the physical means available to the experimenter, or about invisible connections that might exist between the mechanisms involved’

(Pearl (2009), page 364). Kuorikoski (2008) has given insightful discussion of Cartwright’s arguments, distinguishing between effects on variables and parameters. Cartwright may be seen to argue for a process-based viewpoint, in the sense that an intervention initiates a process which we then wish to understand. This is connected to our viewpoint here.

Counterfactual (or potential outcome) models represent another popular approach to causal modelling in addition to the causal DAGs, and the two approaches are in fact related. In the counterfactual setting, an important tool for drawing causal conclusions is to define potential outcomes; different treatment options may have different potential outcomes where one is realized for a given individual whereas the others are counterfactual. A very fruitful theory has been based on this framework which was originally developed, in somewhat different directions, by Donald B. Rubin and James M. Robins; see for example Rubin (1974) and Robins (1986). Counterfactual approaches to causality also play an important role in philosophy; see Woodward (2008). A fascinating review of causal inference based on Rubin’s work is given by Holland (1986) who, interestingly and a little unusually for a statistics text, refers to several major philosophers.

It is interesting to note that a causal DAG may also be defined in a way that does not explicitly refer to intervention; see for example the following definition due to Hernán and Robins (2006), appendix, section 1.2:

‘A causal DAG is a DAG in which (1) the lack of an arrow from node *V*_*j*_ to *V*_*m*_ can be interpreted as the absence of a direct causal effect of *V*_*j*_ on *V*_*m*_ (relative to the other variables on the graph) and (2) all common causes, even if unmeasured, of any pair of variables on the graph are themselves on the graph’.

For this definition to make sense, we need to know what a causal effect is, and a definition is given within a counterfactual setting (Hernán, 2004), although it is not obvious how this definition applies to all the potential arrows of a DAG. In this case the idea of a direct causal effect becomes a primitive concept, as opposed to intervention in Pearl’s definition.

The definition of a counterfactual world is based on a *ceteris paribus* assumption, i.e. a given factor (or set of factors) should be changed and everything else should remain equal. If for instance a person is a smoker, then the counterfactual world might be one in which he does not smoke, but everything else remains the same. Still, this may not be so simple. Consider for example stopping smoking; then it is known that this often leads to other changes, such as an increase in body weight, and the question is how to incorporate this. It may therefore not be obvious how to define the counterfactual world and clearly this is related to the complexity of the intervention concept and the effects of time. An intervention may often be perceived as changing an (approximate) equilibrium into a new (approximate) equilibrium situation with a number of attendant changes taking place. In fact, the interesting paper by Lauritzen and Richardson (2002) connects the concept of a chain graph (an extension of a DAG allowing for undirected edges) to equilibrium distributions in stochastic processes. This gives a promising connection of the causal diagrams to the actual dynamic world that these diagrams will describe.

The great advantage of the causal DAGs and the counterfactual or potential outcome approaches is that they represent systematic and clear views of what a causal effect should mean. Usually in statistics, causality is undefined and we just perform analyses of association and then try to discuss or justify a causal conclusion at the end. Instead, the approaches that were pioneered by Pearl, Rubin and Robins and their co-workers start with a definition of causality and then discuss to what extent causal conclusions can be drawn from the data and how this should be done. This is turning the traditional statistical approach on its head, and that is very useful. Still, as expected, we usually must make untestable assumptions to obtain actual conclusions. Terms such as ‘causal inference’ and ‘causal estimation’ might naively be taken to imply that we have special access to the causal truth, which is clearly not so.

### 2.2. Time is important

Increasingly, statistical analysis is applied to data collected over time. In clinical medicine and epidemiology long-term life histories are becoming available, with repeated measurements and observations at different time points. Often, many processes are observed in parallel for each individual, some containing events and some measurements. For instance, a patient may be monitored regularly with many clinical parameters being measured, with treatments changing several times, and with several clinically important events happening. Also in basic biomedical research time is becoming a relevant parameter. One is, for example, starting to observe gene expressions at consecutive time points, and this may give the possibility of analysing how the genes influence one another. One aim of this paper is to understand the relationship between processes; which processes are driving others? This is expressed well by Wolkenhauer and Hofmeyr (2007) who said

‘…causation as the principle of explanation of change, is a relation, not between things, but between changes of states of things’.

This is also in agreement with the opinion that was voiced by Diggle *et al.* (2007) that analysis of longitudinal data will focus on increments of processes.

Time dynamics are often surprisingly absent from analysis; one may include time, but often just as a tool to produce an overall or marginal effect. Even a major work on causality such as Pearl (2009) does not include much on how developments over time aid the understanding of causality. It is clear that the directions that are embedded in a causal DAG somehow correspond to direction in time, but there is usually no explicit timescale. For example in the discussion of various DAGs in Hernán *et al.* (2002) a collider-type argument is given for why one should not adjust for birth weight when assessing whether low folate intake increases the risk of birth defects. One could alternatively have argued that birth weight is measured late in the process and one would always be wary of conditioning on something measured late in time, especially if it is concomitant to or occurring later than the outcome. This follows from the fact that causality works forward in time. This is also connected to the issue of independent censoring that is discussed below. Colliders play an important role in the DAG theory, but their definition (two arrows pointing to the same node) entails that they will represent events that come after other important events, including possibly the outcome.

There is of course much causal literature where time is included, e.g. Gill and Robins (2001). Another major reference is Robins (1994), who introduced the structural nested mean model where time direction is explicitly included. In this context, structural models mean that they are formulated in terms of counterfactual, or potential, quantities, as opposed to classical statistical models which are formulated as models on actual observations. For a very accessible introduction to the broader class of structural mean models, see Lynch *et al.* (2008). Complications may arise in the case of binary outcomes; see Joffe *et al.* (2007), page 81, and Robins and Rotnitzky (2004). Another example where time comes in is the important marginal structural model (Robins *et al.*, 2000) which we shall return to in Section 4.5. Our emphasis is on understanding the dynamic development over time as opposed to a marginal analysis, and we mostly consider a time continuous situation.

## 3. Direct and indirect effects–mediation

The concept of direct and indirect effects is very important for deeper understanding of causal mechanisms and we shall give a discussion of this. It is related to a mechanistic thinking and the idea of pathway analysis.

As an example, consider the study of Braaten *et al.* (2004) which analysed the increased risk of breast cancer among highly educated women. They showed that education has only an indirect effect on breast cancer, mediated through other established factors, such as parity and age at first birth. Highly educated women, for instance, tend to have children later than those with less education, and this delay is known to be a risk factor. Education in itself, as we might expect, has no direct effect.

It is important to realize that the understanding of what are direct and indirect effects will usually be provisional and dependent on the level of knowledge that we assume. An effect may appear to be direct, but this may just be because we have not measured or do not know about or want to consider the relevant mediating factors. Consider for example the discussion given by Francis *et al.* (2008) where they distinguished direct and indirect effects of osteoporosis treatment on back pain. There is an indirect effect on pain due to a reduction in the incidence of vertebral fractures, but also a possible direct analgesic effect by influence on the central nervous system. Clearly, the direct effect could also be divided into several steps if we had knowledge of the detailed biochemical mechanisms. Hence, in a sense, direct effects do not really exist.

The choice of the level of the biological understanding that is involved is partly dependent on the actual knowledge we have. We may consider an effect as direct because we do not have sufficient knowledge of the detailed pathway and the individual steps that are involved. Still, it could just as well be a matter of the choice of focus. In Francis *et al.* (2008) it appears that one is interested in the particular issue of whether the medication primarily prevents fractures, or also has an analgesic effect, and the terms ‘direct’ and ‘indirect’ effects are used to describe this particular aspect. It may not be relevant to go into a further analysis of the detailed mechanism by which, say, the analgesic effect comes about. Hence, there is generally nothing absolute about the idea of direct and indirect effects, but it depends on the setting and the focus. However, we may also meet these concepts at a rather fundamental biological level, e.g. in transcriptional regulation networks; see for example Shen-Orr *et al.* (2002) and Wagner (2002).

Another aspect of direct or indirect effects is the use for interpretation of causes in a causal network. When asking about the cause of an accident, say, there is usually no particular single cause that can be considered as *the* cause of the accident. One could point out that the driver was drunk, but then one may ask why the driver was drinking. Maybe one cause of this is a genetic disposition for alcoholism. Another cause may have been a difficult childhood. Then again one could ask what were the causes of the difficult childhood etc.

The idea of a causal network is expressed very well in the following quotation from Coggon and Martyn (2005):

‘Any cause of disease will itself have causes. And each of these causes will have causes, in a theoretically infinite causal chain. Acute myocardial infarction is caused by (among other things) atherosclerosis of the coronary arteries, which is caused by (among other things) high plasma cholesterol concentrations, which is caused by (among other things) a high dietary intake of fat, which is caused by (among other things) living in 21st century western society, and so on. In a sense, all diseases have a potentially infinite set of causes and so can never be completely understood.’

This view of causality is one reason why cause is considered such a difficult, and rather woolly, concept. The idea of indirect effects and mediation may serve to tie this unruly causal chain together. Probably, high dietary intake of fat has an influence on atherosclerosis that is mediated through cholesterol, with a possibly direct effect in addition, or with effects mediated through other mechanisms. Some statistical theories of mediation, such as classical path analysis (Loehlin, 2004; Wright, 1921, 1934) as well as dynamic path analysis (Fosen *et al.*, 2006b), give simple prescriptions for calculating effects along such pathways, which hold in linear models, namely to multiply path coefficients along the pathway. If this is applicable, then the bits and pieces of the causal chains can, at least in principle, be connected, and there is no contradiction between many different possible causes in a network.

Statistical analysis of mediation is often difficult. For instance, Cole and Hernán (2002) presented a critical view of the possibility of estimating direct effects, pointing out that the causal status of such estimation may not be assured even in randomized and double-blind trials because of possible confounder effects between mediator and outcome.

There are different possible formulations of direct and indirect effects in a statistical framework. We shall present one suggested in the theory of potential outcomes, and thereafter discuss a dynamic formulation.

### 3.1. Counterfactual definitions of mediation

In statistics it is common to define direct effects by conditioning on some mediating variable; this for example is used in standard path analysis (e.g. Loehlin (2004)). Path analysis is based on a DAG, like in Pearl’s theory, but there is no attempt at a formal causal theory. The DAG represents *a priori* presumed influences between variables and the statistical analysis consists in regressing the variable that is represented by a node on all variables represented by parental nodes. Repeated linear regressions are done. Indirect effects along a certain path are found by multiplying path coefficients (estimated by linear regression) along the path. If two nodes are linked directly, then the path coefficient for this link represents the direct effect.

This is not a proper causal definition as has been emphasized in the causal literature. Pearl (2009), page 132, pointed out that a proper causal definition of the direct effect may be given in terms of his do operator. A more general approach, also incorporating indirect effects, may be based on potential outcomes. An interesting study of this was given by Joffe *et al.* (2007) and we follow their presentation here. They focused on the effect of a treatment *A* on an outcome *Y* via a possible mediator *S*. The direct effect of the treatment on the outcome is defined by controlling for the mediator. This is seen as the contrast between the potential outcomes *Y*(*a*_1_,*s*) and *Y*(*a*_2_,*s*) for two treatment levels *a*_1_ and *a*_2_ when the mediator is set at a given level *s*. The idea is that the mediator can be *physically manipulated* (Joffe *et al.* (2007), page 91) to this given level for both treatment levels. This definition of a direct effect goes back to Robins and Greenland (1992) and was also stated very clearly by Pearl (2000), page 127:

‘…the requirement of holding the mediating variables fixed must be interpreted as (hypothetically) setting these variables to constants by physical intervention, not by analytical means such as selection, conditioning or adjustment’.

This definition is of a controlled direct effect, but one may also find a somewhat weaker concept in the literature, named the natural direct effect (Pearl, 2001; Robins and Greenland, 1992). Instead of fixing the mediator *S* at a specific level, we choose a reference treatment a^*^ and then let each individual have the natural level of the mediator, which is denoted 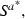 corresponding to this treatment. The natural direct effect is then given by the contrast between the potential outcomes 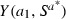 and 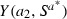, where a^*^ could be either *a*_1_ or *a*_2_.

The natural indirect effect can be defined as the contrast between 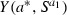 and 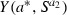; hence the treatment is fixed at some level a^*^ and we then compare the outcomes at the natural levels of the mediators under two treatment levels.

Following Emsley *et al.* (2010), page 250, we make the assumption that the direct effect is independent of the reference treatment; hence we can write it as 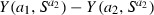. Using *a*_1_ as a reference treatment for the indirect effect yields 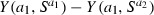. Adding up the direct and the indirect effect yields 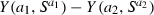, which is exactly the total effect. Note that the quantity 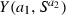 depends on manipulating the mediator to a different value from what it would naturally have under the treatment *a*_1_.

There is no doubt that the counterfactual definition of direct and indirect effects has a very useful clarifying role in many contexts, as for example demonstrated by Emsley *et al.* (2010); see also Petersen *et al.* (2006) for a very accessible introduction. *G*-estimation of the direct effect based on these ideas has been discussed in Joffe *et al.* (2007) and Ten Have *et al.* (2007). Some discussion on interaction between treatment and mediator was given by Joffe *et al.* (2007). Discussion of direct and indirect effects in terms of principal stratification has been given by Rubin (2004).

We shall have a critical look at the empirical meaning of the counterfactual approach in this setting. Both controlled and natural direct effects, as well as natural indirect effects, require manipulation of the mediator to levels that do not correspond to the actual treatment given. Didelez *et al.* (2006) state about the natural direct effect (here *X* is the treatment and *Z* is the mediator):

‘…we need to think of a way of intervening in *X*, changing it around, while *Z* can still be generated as if *X* was kept at its baseline value. For the natural direct effect to be empirically meaningful it needs to be made clear how such interventions can be carried out in practice.’

They pointed out one important case where an intervention of this type can be carried out: often in clinical trials there may be a placebo effect. By blinding the study one presumably achieves that the placebo effect will be the same in each treatment group, and so an estimate of the biological effect of the medication (the direct effect) can be made. There are also other types of experiment which may amount to manipulating a mediator, for instance the blocking of receptors in experiments in cell biology.

Still, in many cases it may be different. As an illustration we introduce an example that will be used later in a more extensive form, namely treatment for HIV infection. The ‘highly active anti-retroviral treatment’ (HAART) is designed to attack the spread of HIV in the body and it usually very efficiently reduces the viral load (although it does not exterminate the virus completely). This effect on the virus is probably the main reason for the great efficiency of HAART as a treatment to delay, or even to stop, progression. Still, there is some evidence that HAART also has beneficial effects that are not mediated through virus suppression, i.e. direct effects in our context (Lu and Andrieu, 2000; Monini *et al.*, 2003; Bernstein and Dennis, 2008). Still, we could hardly imagine giving HAART and then manipulating the viral load to be at the same level as it would have been without treatment as required for a counterfactual definition. Apart from the ethical aspects and practical difficulties of doing this, even if an experiment could be carried out this would probably change the underlying situation and so would be difficult to interpret. What you do instead is to treat people who are not HIV infected with HAART, showing that the treatment has a positive effect on a number of conditions (Monini *et al.*, 2003), or you consider HIV patients who do not respond on HAART with respect to viral load, but whose immune system as gauged by the CD4 cell count still improves. Hence, we collect *circumstantial evidence* for the existence of pathways of the effects of HAART that are not mediated by viral load.

As another example, Goetgeluk *et al.* (2009) discussed various ways to estimate direct effects within the counterfactual framework. Several types of direct effect are mentioned, although the main emphasis in the paper is on the controlled direct effect. Goetgeluk *et al.* (2009) showed how proper estimation can be carried out even when certain types of confounding are present. They applied the theory to a comparison of two methods of subfertility treatment. A difference in birth weight between the two types of treatment was observed and the question is whether some of this effect is due to a difference in gestational age between treatments. The idea is to fix gestational age (the mediator) and to estimate the remaining effect on birth weight. They estimated a controlled direct effect, but again it is not obvious at all that gestational age can be fixed in a meaningful way, and the interpretation of their analysis therefore remains somewhat unclear. Of course we could imagine relevant interventions, such as forcing a birth to start early, on a set date, or possibly attempting to delay a birth, but such an experiment would hardly be carried out in practice unless there were strong medical reasons to do so.

We shall assert that the idea of manipulating the mediator to be uninfluenced by the treatment requires an actual ability to intervene to achieve this. It seems unsatisfactory to base a concept on interventions that may be impossible or inconceivable in practice. Whether interventions must be something that can be carried out by humans is controversial; see Woodward (2008). Still, there is clearly a difference between cases where the mediator can relatively easily be manipulated, such as the education–breast cancer example that was mentioned at the beginning of Section 3, and one where the mediator is determined by physical or chemical laws like in the HIV case. In the education–breast cancer case (Braaten *et al.*, 2004) we could imagine instituting policies, such as generous economic support, that would encourage women to have children early even if they received high education. In this case, we are not up against physical or chemical laws.

Petersen *et al.* (2006) also discussed the HIV example and admitted that there is no corresponding real world experiment to the manipulation that is required for the definition of direct effect, but they still argued that the controlled and natural and direct effects make sense. Interestingly, they say that the purpose is precisely to learn about the mechanism of treatment, but then it is not clear why the definition should depend on external manipulation. They also appealed to the reader who is ‘uncomfortable with a counterfactual interpretation of the direct effect parameters’, giving a more intuitive explanation.

This is not a criticism of counterfactual ideas *per se*; the point is that *nested* counterfactuals may sometimes be undefined in any reasonable sense.

In natural science, experiments are being done all the time, and such experiments are often interventions that elucidate causal connections. This is for instance how one gradually builds up an understanding of mechanisms and pathways in biology. In most cases such interventions have a limited scope and cannot test out all the complexities of a system. In general, experiments and interventions may be seen as tools to learn about or to exploit causality for some purpose. This only works because there is an underlying causal structure which would be present whether we could intervene or not. Intervention and manipulation exhibit causality, but do not necessarily define it.

### 3.2. Mechanistic interpretation of mediation

It is natural to think that mediators are actual physical mechanisms. Their existence is not dependent on whether we can imagine manipulating the mediators or not. These mechanisms describe something that actually takes place, say in a human body. Such a mechanistic view of mediation was forcefully presented by Baron and Kenny (1986), page 1173. They defined mediation as

‘the generative mechanism through which the focal independent variable is able to influence the dependent variable of interest.’

The idea of a mediator plays a large role in biology; for example about 2000 papers were found (at the beginning of year 2010) in the single journal *Nature* when searching for ‘mediator’ and many of these appear to be mediators in a pathway sense implying a mechanistic understanding. The scientific evidence behind such mediators consists of various types of experiment and laboratory studies coupled with basic biological knowledge.

In fact, Pearl himself briefly indicated that manipulation may not be a panacea for understanding causality (Pearl (2010), page 42, and Pearl (2009), section 11.4.5) and made the interesting statement ‘The mantra ‘‘No causation without manipulation’’ must be rejected’. Rather, Pearl indicated, causality could be understood in terms of signals passing through and being sensed in the system. This has interesting connections with the emphasis on signalling that pervades biology today.

The concept of a pathway diagram plays an increasingly important role in biology and other fields, especially with the present growth of systems biology. The pathway diagram is a way of describing mechanisms. In biology we may consider that the effect of a substance (e.g. a hormone or medication) is transmitted, by biochemical reactions, through a number of paths in a possibly very complex network. A node in such a network would be a mediator if a path passes through this node. Hence the mediator has a physical meaning in such a network, and we consider that direct and indirect effects, and mediators, give some kind of description of real processes unfolding in time. The indirect effect passes though the mediator whereas the direct effect does not.

Let us return to the example of HIV infection. HAART is a mixture of several types of medication that attack the replication of HIV in various ways. This is expected to lead to an improvement for the patient, measured for example by the CD4 cell count. This is what is also observed and hence we would expect a major part of the treatment effect to be mediated by the reduction of viral load. This is a mechanistically based concept of mediation.

In fact, the complexity of many pathway systems published in biology is very striking; often there is a very large number of nodes and connections between them. Examples of such pathways are abundant in the biomedical literature and on the Internet.

In fact, the complexity of many pathway systems published in biology is very striking; often there is a very large number of nodes and connections between them. Examples of such pathways are abundant in the biomedical literature and on the Internet.

In climate research pathway ideas are also used. Kaufman *et al.* (2002) studied the effect of aerosols on warming, defining the concept of the aerosol direct effect (reflecting and absorbing solar radiation) and the aerosol indirect effect (cooling the surface). The proper understanding comes through a combination of the direct and indirect pathways. Considering more generally the emission of carbon dioxide into the atmosphere it is clear that this triggers complex sets of reactions through many different pathways which are only partly understood.

### 3.3. Connecting mechanisms, causality and statistics

The use of the causality concept in relation to mechanisms is a matter of dispute. Some will say that mechanism is not the same as causality. The concept of mediator seems to be clearly a mechanistic concept as shown in the discussion above. Still, it should be pointed out that direct and indirect effects are in a somewhat different position. These effects concern what happens when a mechanism is ‘turned on’, i.e. there is some input which produces an output. Still, if we have a detailed description of the mechanism as a function of time, then we can (in principle) compute the various effects that come from an input and the various paths that these effects are taking. Therefore, a good mechanistic understanding may imply a causal understanding. It is also important to realize that a causal concept like ‘influence’ could either mean that there is an intervention from outside, or it could describe an internal relationship, e.g. when the amount of HIV in the blood influences the status of the immune system as measured by the CD4 cell count (Section 6).

One statistical formulation of causality that is closely related to a mechanistic view is the Granger causality concept (Granger, 1969) which originated in a suggestion by Norbert Wiener. Granger gave detailed mathematical formulations of his causality concept in a time series setting, where the point is to model the influence between processes locally in time. Intuitively, if the change in a process *X* at any given time is dependent only on the past history of *X* itself and not on some other process *Y*, then we say that *X* is not caused by *Y* in a Granger sense. The idea is similar to the local independence concept that was discussed in Section 4.3. Superficially, Granger causality is a purely statistical concept, defined essentially by some type of conditioning. For example spurious local (in)dependence may exist in cases where important processes are not included. Granger was entirely aware of this and included ‘all the information in the universe’ in the conditioning (Granger, 1969) to justify calling it causality. The idea is that one shall have included sufficient processes and information, also unmeasured confounder processes, so that the model is a description, at some level, of a mechanistic relationship between physical processes. One great advantage of Granger causality is that time is playing a major role. Direct and indirect effects can also be defined within this framework (Eichler, 2007).

Granger causality was developed in economics but also plays an increasing role in other fields, e.g. for determining mechanisms in neuroscience (Wang *et al.*, 2007). An important area where causal analysis of pathways is being used is the field of brain connectivity. A stimulus is given to some area in the brain and one then analyses how the response spreads to various parts of the brain by using functional magnetic resonance imaging (Londei *et al.*, 2006; Friston, 2009). This is a model for the actual spread of signals in the brain between different areas and direct and indirect effects are apparently understood in this physical sense. The tools for analysis are Granger causality and related approaches. For a very interesting application of Granger causality to the issue of climate change, see Elsner (2007) who studied the occurrence of Atlantic hurricanes. One should realize that Granger causality has generated a much applied practical approach which reveals mechanistic relations in many fields.

Another type of model which is relevant for understanding pathways is the structural equation model. This idea was developed by Haavelmo; see for example Girshick and Haavelmo (1947) for an excellent introduction. The structural equation model, which by the way was a major inspiration for Pearl (2000, 2009), is intended as a model for actual mechanisms. The model may be expressed in terms of equations which are superficially similar to regression equations. The major difference from a purely statistical regression model is that parameters in one equation may change, because of a change in policies, without any changes occurring in other equations.

An extension of structural equations to processes developing over time would be the dynamic systems. In physics and other fields these are typically used to decribe mechanistic relationships and are often modelled by differential equations. In biology there would be a correspondence to pathways. There is large literature on dynamic systems; see for example Commenges and Gégout-Petit (2009) which gives a very general and broad introduction to a system view of causality. It is natural to think that a flow of information (Granger, 1969) or signals pass through the system along various paths.

Now our modest suggestion is as follows: if a direct effect cannot reasonably be defined as a controlled or natural direct effect in the counterfactual sense because the required hypothetical manipulation of the mediator is inconceivable, then we can alternatively view these effects as being represented by flow in a dynamic system, so that the direct effect corresponds to the flow not passing through the mediator. The indirect effect could similarly be understood as that passing through the mediator. Some precision can be made to this concept in a mathematical setting.

## 4. Mathematical formulations of pathways

Whereas the Bayesian networks have a precise mathematical formulation, the situation is more complex for pathways, which may not simply be associated with one single formulation. In the literature there are different mathematical approaches for modelling pathways. Some formulations focus on a network system, characterizing various aspects of this. Other formulations concentrate on differential equations, which in fact were developed to track dynamic systems developing over time. Other models, again, are of a stochastic nature. We shall consider just a few simple models of the possibly large number of conceivable models.

We would like to point out that there are two pertinent issues when viewing causality in a mechanistic sense:

(a) time direction–the past influences the present and the future;
(b) locality–the effects are transmitted continuously in time.

The approaches that we discuss below take care of both these issues. We start with differential equations. These are very different from the regression equations of statistics, since setting up a differential equation would almost always be based on some structural insight.

### 4.1. Differential equation models

As suggested by Wolkenhauer *et al.* (2005) differential equations may be useful for modelling pathways. A very simple example of a differential equation model for an output vector *X*_*t*_ would be a linear system of the kind 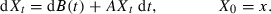
1

This means that the change of *X*_*t*_ in a short interval (*t*,*t*+dt) is determined by the external input vector d*B*(*t*), and by internal input from *X*_*t*_ itself through the matrix *A* (an example of this is given below). Many models will tend to be non-linear, which enables stabilizing feedback effects.

A more realistic picture of biological processes arises if we also include stochasticity. The system in equation (1) can be extended by including a stochastic contribution as follows:


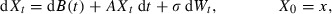
2

where d*W*_*t*_ denotes a white noise process that introduces a random disturbance to the development of *X*_*t*_. (*W*_*t*_ is a Wiener process; see for instance Aalen *et al.* (2008) for a brief introduction to this process and for a discussion of what the ‘derivative’ d*W*_*t*_ means in this context.) The influence relationships are defined in terms of the matrix *A* just as for the differential equation model.

Friston (2009) presented a dynamic causal model based on a set of differential equations for brain connectivity, where ‘causal’ refers to a mechanistic view. This is an interesting attempt to capture how activity in one brain area influences activity in another, i.e. to understand the mechanisms, or pathways, at some level. Similar models were studied by Londei *et al.* (2006).

**Figure 2:**
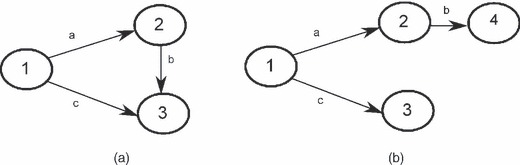
(a) Relationship between components of a stochastic differential equation and (b) transformation made for calculating direct and indirect effects

We may define intervention as setting certain elements of the matrix *A*(*t*) equal to 0, such that one of the processes is not dependent on any other. An example would be to put *a* equal to 0 in the matrix (3) below such that the arrow from node 1 to node 2 in Fig. 2(a) disappears. This is in direct correspondence to Pearl’s intervention concept, extending it to an infinitesimal setting. This is related to the concept of a dynamic path diagram presented in Section 5.1.

### 4.2. Direct and indirect effects

We shall consider only linear systems. We shall think of the effect along a path as the expected changes in the output along the path when the input is changed by a certain amount. We partly follow suggestions by Eichler (2007) of defining direct and indirect effects in a Granger causality framework. A very detailed analysis for longitudinal data was given by Cole and Maxwell (2003). They illustrated how the computation of indirect effects requires calculating along all different paths in the system. This is achieved below by a splitting method.

The output along a path is computed by representing the model in a tree structure, so that each path is one branch, and then we do an explicit computation for this new system. This will be illustrated by a simple example that is presented in Fig. 2; note that the nodes in this example are stochastic processes defined by stochastic differential equations as above. Assume that *A* is defined by the following matrix corresponding to Fig. 2(a):


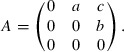
3

Assuming now that the stochastic differential equation can be viewed as structural equations, yielding a true mechanism, we can compute direct effects of 1 on 3, and indirect effects going via 2. For this computation we split process 3 in two as is done in Fig. 2(b) so that the direct effect corresponds to the effect on the new process 3, whereas the indirect effect corresponds to the effect on process 4. The point of this splitting is to be able to distinguish each single path. The *A*-matrix for the new system is denoted A^*^. Assuming *B*(*t*)=0 in equation (2) it follows from Karatzas and Shreve (1998) that *E*(*X*_*t*_|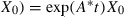 where exp denotes the matrix exponential function. This can be computed by using for example Mathematica (Wolfram Research), yielding a direct effect of *ct*Δ and an indirect effect of 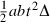 if 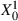 is changed to 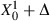. These expressions are supposed to represent the actual flow in the system.

The splitting of states as applied above can be used for more complex systems to follow individual pathways. The splitting may possibly be viewed as some kind of manipulation, but it does not imply setting mediators to different values from what they would naturally have. However, it works only when the actual mechanism is truly a linear system.

### 4.3. Local independence models

We shall now introduce a framework which generalizes the notion of stochastic differential equation. We shall apply the theory of Doob–Meyer decompositions of stochastic processes; see for example Aalen *et al.* (2008) where the background of the present material may be found. The intuition behind this decomposition is quite simple. We first introduce the concept of a *history*, which is the family of events that can be decided to have happened or not happened by observing the past. The history at time *t* is denoted by 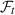. We say that a process *X*={*X*_*t*_} is *adapted to* a history 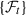 when the following condition holds: if the history is completely known at time *t*, then *X*_*t*_ is also known at this time.

The Doob–Meyer decomposition expresses *X*_*t*_ as a sum of that which is known before time *t*, and the innovation (i.e. new random component) that comes at time *t*. A more formal formulation of this is that under broad conditions a stochastic process can be decomposed uniquely as *X*=Λ+*M*, where Λ is a predictable process, which is often denoted the compensator of *X*, and *M* is a mean 0 martingale. ‘Predictable’ means ‘known from the past’, and the martingale is a sum of random noise (innovations). In many important cases the compensator has a derivative *µ*(*t*)=Λ’(*t*). The process *µ*(*t*) is denoted a local characteristic: a measure of expected local change of the process as a function of the past history.

The local characteristics are ‘drivers’ for the processes, e.g. drift functions for diffusion processes. An important special case is when *X*_*t*_ is a counting process, i.e. counting events occurring. Then the local characteristic is simply the intensity process which gives the rate of a new event occurring as a function of the past history.

A useful concept is the idea of local independence that was introduced by Schweder (1970), extended by Aalen (1987) and then developed further by Didelez (2006, 2007). To obtain a clear understanding of the intuitive content of local independence one may consult references focusing on simple Markov models like Schweder (1970) and Aalen *et al.* (1980), the latter analysing a medical data set concerning whether the menopause influences the risk of the skin disease *pustulosis palmo-plantaris*.

We shall give a more general definition here. Let 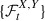 be the history that is generated by the processes *X*_*t*_ and *Y*_*t*_ and let 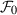 contain information on other random events or influences. Under some general conditions the two processes can be represented by Doob–Meyer decompositions defined with respect to the combined history of 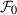 and 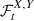:


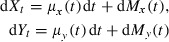
4

where the *µ*s denote the local characteristics of the two processes and the *M*s are martingales. The ‘differentials’, say d*M*_*x*_(*t*), of the martingales are the innovations, representing the new and ‘surprising’ changes in the process. Note that the local characteristics will depend on the history that is being considered.

For orthogonal martingales, define *X*(*t*) to be locally independent of *Y* at time *t* if *µ*_*x*_(*s*) is only a function of *X* up to time *t* (and possibly of some extraneous events), but *not* a function of *Y*. If *X*(*t*) is not locally independent of *Y* it is locally dependent. More formally, let 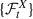 be the history that is generated by the process *X*(*t*); then we have the following definition (Aalen, 1987).

**Definition 1.** Assume that *M*_*x*_(*t*) and *M*_*y*_(*t*) are orthogonal. Then *X*(*t*) is locally independent of *Y*(*t*) if *µ*_*x*_(*t*) is adapted to the combined history of 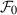 and 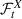.

Clearly, local dependence is an asymmetric concept, it may be one sided (only one process being dependent on the other) or it may indicate a mutual dependence.

The orthogonality between martingales that is assumed in the definition is important. We say that the processes *X* and *Y* are autonomous when the martingales arising from the Doob–Meyer decomposition are orthogonal. The intuitive content is that the innovations (martingale differentials) driving the two sets of processes are different. If this was not so, we could say that *X* and *Y* are partially the same process, which is different from causality. The idea is that processes may be related for (at least) two different reasons: either because they measure partially the same phenomenon, or because they are different phenomena, but such that one has a causal influence on the other (which may be a two-way, i.e. a feedback, mechanism). To take a medical example: an asthma patient may have chronic cough, and also wheezing in the chest. These will be associated, but possibly not in a causal sense since they represent the same phenomenon. However, smoking may cause lung disease, these being two autonomous processes with a causal relationship. Orthogonality is one way of formulating this idea of autonomous processes, but it is not likely that this formulation contains everything that we might want to consider when talking about autonomous phenomena.

Particular applications of the local independence concept are as follows.

(a) When *X*(*t*) and *Y*(*t*) are counting processes, then *µ*_*x*_(*t*) and *µ*_*y*_(*t*) are intensity processes (Aalen *et al.*, 2008). Local independence simply means that the intensity process of *X*(*t*), based on the complete history, does not include information on *Y*(*t*) or its past. The intensity process is defined with respect to the entire observed past. Autonomy in this case simply means that the two counting processes do not jump simultaneously.
(b) When equation (4) models diffusion processes, then the martingales are Wiener-type processes and autonomy means that these processes are independent. The local characteristics are drift parameters in the processes.
(c) If *X*(*t*) and *Y*(*t*) are longitudinal processes measured at discrete times, we may consider time discrete versions 
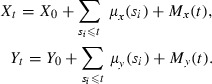
  In this case, the decomposition is simply denoted a Doob decomposition. Local characteristics for longitudinal data were studied by Farewell (2006) and Diggle *et al.* (2007).

Local independence is closely related to Granger causality; see the extension of this concept by Comte and Renault (1996) and Florens and Fougère (1996). The combined concept of Granger–Schweder causality was introduced by Aalen and Frigessi (2007); see also Aalen *et al.* (2008).

So far, we have just considered two processes, but the concept of local independence may easily be extended to several processes. We often use the term ‘conditional local independence’ to indicate that we condition with respect to a past that may contain more than the processes that are explicitly considered. A very general definition of local independence was given by Didelez (2006), and we shall just indicate the intuitive content. Consider for example three groups of processes, *A*, *B* and *C*, and let 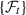 be the history that is generated by all these processes. Then the processes in *B* are locally independent on the processes in *A* given those in *C* if the following condition holds: the compensators with respect to the history 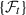 of processes in *B* are adapted to the (more limited) history that is defined by all processes in *B* and *C*. This means that the processes in *A* do not influence the development of those in *B*.

Local independence graphs can be drawn as suggested by Schweder (1970) and Didelez (2006, 2007): an arrow is drawn from node *l* to node *k* if and only if the process corresponding to node *k* is conditionally locally dependent on the process corresponding to node *l*. Local independence graphs are not necessarily DAGs because they may have feedback effects, i.e. there may be mutual local dependence between processes.

In model (2) the local (in)dependence relationships can easily be read off from the matrix *A*, such that for instance 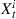 is conditionally locally independent of 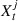, given the remaining processes, if *A*_*i*,*j*_=0. As shown in Fig. 2(a) the particular matrix (3) implies that process 2 is locally dependent of process 1, and process 3 is locally dependent on both 1 and 2. Process 1 is locally independent of both the others, whereas process 2 is locally independent of process 3.

**Figure 3:**
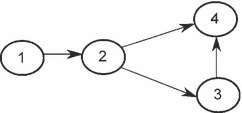
Illustration of direct and indirect effects: node 1 has an indirect effect on nodes 3 and 4, with node 2 as a mediator; node 2 has both a direct and an indirect effect on node 4 with node 3 as a mediator

A simple illustration is given in Fig. 3, where each node represents a stochastic process. Here, process 1 is locally independent of all other processes, process 2 is locally dependent on process 1, but independent of the others etc. For instance, process 1 could be the injection of carbon dioxide into the atmosphere from man-made sources, whereas the other nodes could indicate natural processes that are influenced by this. In the example of Francis *et al.* (2008) node 1 could indicate the decision to take medication, node 2 could be the actual intake of the medicine, node 3 could be the influence on vertebral fractures and node 4 could indicate pain. The graph is supposed to illustrate an actual process, although in a very coarse manner leaving out all the biological detail. Clearly, a much more detailed pathway could be defined in principle, but this is a matter of what is practical for the issue at hand, and also of what is actually known about the underlying process.

### 4.4. Causal status of local independence diagrams

Local independence is defined in a purely statistical fashion but, just as argued in Section 3.3 for Granger causality, it can be given a causal interpretation when all relevant information is included. Hence it is presumed that the processes with their local (in)dependence relationship yield a structural model. The fact that the concepts that are discussed here pick up time local effects and respect time direction is one reason why they may describe mechanisms. As seen, for instance, from Fig. 3 there is a natural interpretation of mediation. Node 1 has an indirect effect on nodes 3 and 4, with node 2 as a mediator. Node 2 has both a direct and indirect effect on node 4 with node 3 as a mediator. More generally we could say that for a local independence graph node *i* is a mediator between nodes *j* and *k* if there is a path from *j* to *k* passing through *i*. In this case there is also an indirect effect. A direct effect is present if there is an edge linking two nodes. Other examples of mediators are shown in the figures of Section 4.5 where, for instance, *L* is a mediator from *X* to *Y*. This way of looking at mediation does not presuppose any ability to manipulate the mediators as required in the counterfactual definition of direct and indirect effects since the pathway model is supposed to describe at some level a presumed physical process.

From a practical point of view there is no major difference between local independence graphs and DAGs as regards their causal status. If you work in a field where experiments can rarely be done, like in observational epidemiology or social science, the way to set up a causal DAG will be to try to include all relevant variables, observed and unobserved confounders, and selection effects and to make assumptions about their causal relationship. This is also a consequence of the definition of a causal DAG that was given by Hernán and Robins (2006), appendix, section 1.2, which was quoted in Section 2.1. Construction of the DAG must be done by subject matter knowledge and judgement. Exactly the same holds for local independence graphs. In practice we must usually make a set of assumptions, some of which will not be testable from the data and there will be arbitrary elements. The difficulties of putting up a realistic causal graphical model have been discussed by Freedman (2004). Nevertheless, both causal DAGs and local independence graphs can be useful tools in the attempts to understand causality.

If you work in a laboratory setting where experiments can readily be done, you have far more opportunity for intervening and thereby assessing the real causal status of various parts of the model. This again goes for Bayesian networks, local independence models and other models. It is important to note that in biology realistic descriptions are often so complex that only parts of the system can be effectively tested by intervention; consider the growth of modern systems biology.

We assert that local independence is a natural concept (but clearly not the only way) for understanding how processes, e.g. in biology, influence one another. Both time direction and locality are taken care of. The idea is that certain processes drive other processes; this is a causal understanding in a mechanistic sense.

There is also a connection to intervention-based causality. Comments on the causal interpretation of local independence diagrams were given by Didelez (2008). An extensive discussion of interventions in connection with local independence is presented in Eichler and Didelez (2010). They used local independence structure to identify causal effects from interventions by using extensions of Pearl’s backdoor and frontdoor criteria.

### 4.5. Formulating confounding and censoring

One major advantage of the causal Bayesian networks, as described by Pearl (2009), is that they may be used for describing the effect of confounders and selection. Similar possibilities hold for the local independence graphs; see Didelez (2008) and Røysland (2011). According to Arjas (2012), local independence is an important concept for controlling potential confounding in causal inference from event history data.

One may for instance define a counting process with censoring, which is of course a type of selection. A standard assumption in survival analysis is that of *independent censoring*. This means that the development of a process does not depend on whether it is censored or not; for a practical discussion, see Aalen and Gunnes (2010). Independent censoring, in fact, means that all other processes are locally independent of the censoring process. To see this, consider the definition of independent censoring that was given in Aalen *et al.* (2008), equation (2.54). We must then consider two histories, namely the history 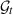 that is defined by the joint model of all (observed and unobserved) counting processes and censoring processes (meaning processes that are 1 when observation takes place and 0 otherwise), and 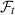, which is the history of all (observed and unobserved) counting processes without censoring. The definition of independent censoring is


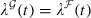
5

where *λ* denotes the intensity of the counting processes with respect to the respective histories. Since the history 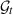 incorporates 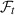 this means that the counting processes are locally independent of the censoring processes. The opposite need not be true; it may be that the censoring processes are dependent on the counting processes.

A very similar formulation may be applied to confounding; see for example Keiding (1999) and Arjas (2012). Use the same set-up as above but, instead of censoring, let the history 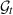 be defined by the counting processes as well as covariate processes. Then the formulation (5) means that covariate processes are not confounders for the counting processes. In this case, the counting processes are locally independent of the covariate processes, but the opposite need not be true; it is quite possible that the counting processes influence the covariate processes. Obviously, the same formulation is valid for any process that is governed by a local characteristic.

**Figure 4:**
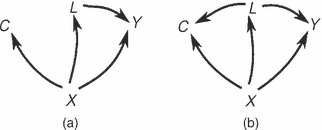
Local independence graph showing how the treatment process *X* influences the outcome process *Y*, the observed confounder process *L*, and the censoring process *C*: (a) independent censoring; (b) process *L* influences *Y* and *C*; all other processes are locally independent of the censoring process

Fig. 4 illustrates a situation with an outcome process *Y* where the censoring process *C* is locally dependent on the treatment *X* (which could be a process) in Fig. 4(a), and also on the mediator process *L* in Fig. 4(b), and where there are no unmeasured confounders. All processes are locally independent of *C* in both Fig. 4(a) and Fig. 4(b). The situation in Fig. 4(a) corresponds to independent censoring whether *L* is observed or not. In Fig. 4(b) the censoring is dependent when *L* is ignored, like in a marginal model, or when *L* is an unmeasured confounder. Røysland (2011), theorem 2, shows how we can come from the situation in Fig. 4(b) to that in Fig. 4(a) by an appropriate weighting procedure, with the dynamics of the remaining processes being kept unchanged. This is a time continuous mathematical formulation of the inverse probability weighting procedure; see for example Robins *et al.* (2000). If *L* is considered as a covariate process, it would be of the type of an internal, or endogenous, covariate process and it requires great care to distinguish between direct and indirect effects. However, under independent censoring, the total or marginal effect is found simply by ignoring *L* and using standard survival analysis.

The concept of *d*-separation is well known from the theory of causal DAGs (Pearl, 2009). There is an analogous concept for local independence graphs called *d*-separation which was developed by Didelez (2008). This is defined as follows: consider a directed graph and let *A*, *B* and *C* be three disjoint subsets of nodes. We restrict ourselves to the ancestral graph that is generated by *A*, *B* and *C*, i.e. the part of the graph with nodes in *A*, *B* and *C* as well as their ancestors. Next, delete all directed edges starting from *B*. Further, consider the moral graph (Lauritzen and Spiegelhalter, 1988), i.e. the undirected graph where the parents of all children are ‘married’, i.e. a connection is drawn between the respective nodes. (The nodes that point to a node *Z* are called the parents of *Z* and are denoted pa(*Z*), whereas the nodes to which there is an arrow originating in *Z* are called the children of *Z* and are denoted ch(*Z*).) Then *Cd*-separates *A* from *B* if it graphically separates *A* and *B* in the moral graph. Didelez (2008) showed how *d*-separation can be used to factorize likelihoods, just like *d*-separation for DAGs.

In Fig. 4(a)*C* is *d* separated from *Y* by *X*. The ancestral graph is precisely the graph and no outgoing edges from *Y* must be deleted. There are no ‘unmarried’ parents, so no edges need to be added by moralizing. Since the only connection between *C* and *Y* goes through *X*, we have proven *d*-separation.

**Figure 5:**
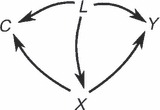
Local independence graph showing how the treatment process *X* influences the outcome process *Y* and the censoring process *C*; the confounder process *L* influences *X*, *Y* and *C*

The definition of time-dependent confounding is also readily interpreted within this framework. In Fig. 5 the process *L* is a confounder of the treatment process *X*, the censoring process *C* and the outcome process *Y*. In this case *L* is an external, or exogenous, time-dependent covariate and correct analysis of the effect would be achieved for instance by a Cox model with time-dependent covariates.

**Figure 6:**
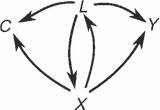
Local independence graph showing how the treatment process *X* influences the outcome process *Y*, the confounder process *L* and the censoring process *C*: the confounder process *L* influences *X*, *Y* and *C*

A more general graph is given in Fig. 6 where there is time-dependent confounding between treatment *X* and the covariate process *L*. This is a situation which has been very thoroughly studied in connection with the analysis of treatment for HIV infection (Hernán *et al.*, 2000; Sterne *et al.*, 2005; Gran *et al.*, 2010). In that case treatment is only started when the CD4 cell count, which is an indicator of the status of the immune system, declines below a certain level. It has been demonstrated that standard survival analysis gives a biased analysis, but that a marginal structural model yields correct results (Hernán *et al.*, 2000). A detailed analysis of the marginal structural model using local independence and *d*-separation is given by Røysland (2011). He shows how inverse probability weighting procedures, which are akin to the marginal structural model, might bring us from the situation in Fig. 6 to that in Fig. 4(a). It is demonstrated how local independence is precisely the concept that is needed to disentangle the causal issues in a precise mathematical framework. Note that the graph in Fig. 6 is not a DAG; typically feedback will produce non-DAG elements in local independence graphs.

## 5. Dynamic path analysis

The Bayesian networks of the now classical causal DAGs may seem quite separate from the dynamic models that are described here. In fact, there is a way of connecting these concepts. The idea is to focus on the outcome in a small time interval, defined as the change in a process. Examples of such outcomes are

(a) the differential of a (possibly stochastic) differential equation (for example in the linear system of equation (1) we could focus on 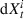 as the outcome at time *t*; this differential is a linear combination of the components of *X*_*t*_),
(b) single jumps in a counting process and
(c) increments in a time discrete longitudinal process (Diggle *et al.*, 2007).

From equation (1) we may write 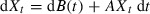. This can be seen as a differential version of a regression model, where the differentials are the outcomes. We want to generalize this.

Graphical models may be defined in this time local setting, just as well as for ordinary random variables. Dynamic path analysis consists of a particular diagram and a statistical method for analysing the relationship. The dynamic path diagram is a DAG similar to those used in classical causal inference. Still, there are two major differences. Firstly, the dynamic path diagram is defined as a function of time, and path coefficients may change over time. Secondly, the outcomes in the diagram are increments in stochastic processes. This is a time-localized version of a causal diagram, being itself a stochastic process. An example could be that the outcome is the jump in a counting process, whereas the other variables in the diagram are covariates that influence the outcome. These may be either fixed, dynamic or time-dependent covariates; see Aalen *et al.* (2008) or Fosen *et al.* (2006b).

Dynamic path analysis can be used for example as a tool for assessing direct and indirect effects according to the pathway definition. The effect of confounders and other biasing effects can be evaluated by the same tools as for causal Bayesian networks.

### 5.1. Dynamic path diagram

A dynamic path diagram is a set of DAGs *G*(*t*)=(*V*(*t*),*E*(*t*)) indexed by time *t*. Here *V*(*t*) denotes the vertices and *E*(*t*) the edges at time *t*. At any time *t*, the vertex set *V*(*t*) is partitioned into a *covariate set*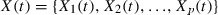 and an *outcome process*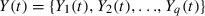. All processes are adapted to a history 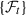, and covariate processes are assumed to be predictable. We shall assume that the vertices in the graph as well as the partition into covariates and outcomes are time invariant. However, the edge set *E*(*t*) may vary with time. We shall assume that any set of edges respecting the DAG assumption is allowed, except edges pointing from an outcome to a covariate. The components of the outcome process *Y*(*t*) will have no children.

Note that the set of diagrams is a stochastic process. The outcome process *Y*(*t*) is represented in the diagram through its increment or differential (in a general sense, including possible jumps, or time discrete increments). The covariate process may have unmeasured components, assuming possibly the role of unmeasured confounders.

**Figure 7:**
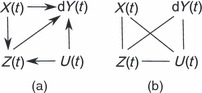
(a) Dynamic path diagram with exposure *X*(*t*), mediator *Z*(*t*), outcome d*Y*(*t*) and unmeasured confounder *U*(*t*) and (b) moral graph corresponding to a lack of direct effect from the exposure *X*(*t*) to the outcome d*Y*(*t*): it is seen that the mediator *Z*(*t*) does not block the path between *X*(*t*) and d*Y*(*t*), but that there is also a path through the unmeasured confounder *U*(*t*)

A simple example of a dynamic path diagram is given in Fig. 7(a). Here we have an exposure *X*(*t*) which influences a mediator *Z*(*t*) and an outcome d*Y*(*t*). The diagram includes a possible unmeasured confounder *U*(*t*). In the terminology above, the covariate process includes *X*(*t*), *Z*(*t*) and *U*(*t*). Note that all variables must be a function of what has been known before *t*, apart from d*Y*(*t*) which measures the increment (or differential) at time *t*. We assume that time discrete processes are also included, in which case d*Y*(*t*) would be the increment over the last time interval (the interval ending in *t*). Moving on from time *t* to a later time implies updating of the variables that are involved in the diagram.

The covariate processes may be observed in parallel with the outcome process, or they may in some cases be actual functions of the outcome process. For instance, if *Y*(*t*) is a counting process, then *Z*(*t*) could be the number of jumps in the counting process from time 0 and up to (but not including) time *t*. The covariate processes represent the past influencing d*Y*(*t*) and usually will be summary measures that are thought to pick up the main influential features of past development. For time-dependent covariates it will often be a question how they influence the further development. Is it the present value that is most important, or is it the recent changes in the covariates, or possibly earlier values? In practice, we must make relatively simple models picking out main features.

Fig. 7(a) may be viewed as a Bayesian network. As discussed in Section 5.2 linear regression is usually performed with d*Y*(*t*), so that we shall normally have a linear system. The considerations in Pearl (2009) concerning confounding and *d*-separation will be valid for any given time *t*. The calculation of the joint distribution on the basis of a Markov structure can be done as for Bayesian networks with the following exception: the conditional probability distribution of the outcome is substituted by the conditional local characteristic of the outcome process. Note that the Markov structure here is to be understood in the graphical sense, and not with respect to time.

Let us consider a general dynamic path diagram with just one outcome node and let the outcome process be a marked point process, i.e. the combination of a counting process *Y*(*t*) with a mark *X*(*t*) associated with each jump, the mark in this case being the set of variables defined by the nodes in the DAG, apart from the outcome node. Consider the product of the mark-specific intensity process of *Y*(*t*) and the probability distribution of *X*(*t*), i.e. *λ*(*t*|*X*) *P*_*t*_(*X*). The joint distribution over all nodes combined with the mark-specific intensity process is 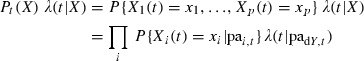


where pa_*i*,*t*_ are the Markovian parents to the covariate process *X*_*i*_(*t*) (i.e. the subset of covariate processes that are parents within the graphical model), whereas pa_d*Y*,*t*_ consist of the parents of d*Y*(*t*). The probability expressions, such as 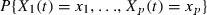, are generic, meaning actual probabilities in the discrete case and densities in the continuous case.

When viewing dynamic path diagrams from a causal point of view, this can be done in the sense of causal DAGs as discussed in Section 2.1. If we assume Pearl’s interventional point of view, then a series of interventions must be considered, one for each relevant time. Alternatively, we may view dynamic path diagrams as a tool for assessing local (in)dependence, which will correspond to a causal structure if sufficient information and confounders are included; see Sections 3.3 and 4.4.

Returning to the example, assume that there is no effect of *X*(*t*) on d*Y*(*t*), which is relevant when assessing local independence. Using a linear model, there should be a zero regression parameter in equation (6) in Section 5.2. To judge the validity of the regression, we must consider the issue of confounding. It is well known that there may be a problem if there is an unmeasured confounder process. This can be seen by a standard argument, using the moral graph which was defined by Lauritzen and Spiegelhalter (1988), and is an alternative to using *d*-separation. The moral graph was briefly defined in Section 4.5. Firstly, we consider the ancestral graph of *X*(*t*), *Z*(*t*) and d*Y*(*t*), which will consist of all four nodes in Fig. 7(a). The moral graph, as shown in Fig. 7(b), shows that it is not sufficient to condition with respect to the observed covariate *Z*(*t*) when doing the estimation, since there is also a path through the unmeasured confounder *U*(*t*). This is a well-known problem for this type of models.

**Figure 8:**
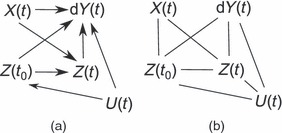
(a) Dynamic path diagram with exposure *X*(*t*), covariate *Z*(*t*_0_), mediator *Z*(*t*), outcome d*Y*(*t*) and unmeasured confounder *U*(*t*) and (b) moral graph corresponding to a lack of direct effect from the exposure *X*(*t*) to the outcome d*Y*(*t*): it is seen that the covariates *Z*(*t*_0_) and *Z*(*t*) block the path between *X*(*t*) and d*Y*(*t*)

When variables, e.g. mediators, are measured repeatedly over time, the situation may be different. In Fig. 8(a) we assume that the covariate process is first measured at some *t*_0_ before treatment, yielding the value *Z*(*t*_0_), and then after treatment with the value *Z*(*t*). Then the effect of the exposure is connected only to *Z*(*t*). There might be an unmeasured confounder that connects the covariate process with the outcome. However, it is not unreasonable to assume that the confounder effect can be seen as an effect on *Z*(*t*_0_) in the model, with the effect on *Z*(*t*) being only through *Z*(*t*_0_). Assuming no effect from the exposure *X*(*t*) to the outcome d*Y*(*t*) yields the moral graph that is seen in Fig. 8(b). We see that all paths from *X*(*t*) to d*Y*(*t*) are blocked by the observed variables *Z*(*t*_0_) and *Z*(*t*), and hence the effect of *X*(*t*) on d*Y*(*t*) can be estimated.

Assume now that we wish to interpret the effect from *X*(*t*) to d*Y*(*t*) as a direct effect and the effect going via *Z*(*t*) as an indirect effect. We may attempt to define controlled and natural direct effects for this case, but note that the time aspect here may complicate issues. Developments of these ideas under certain limiting assumptions have been made by Lange and Hansen (2011), who estimate natural direct and indirect effects, and Martinussen *et al.* (2011), who estimate controlled direct effects by using *G*-estimation. More work is needed in this area. Our alternative view is again to consider the model as describing at some level a dynamic system, such that the estimates describe the actual movement of signals, or influence. In both cases we must require, essentially, that the treatment–outcome relationship and the mediator–outcome relationship are unconfounded, and that there are no interactions between the treatment and the mediator (see also Martinussen (2010), section 2, and Cole and Hernán (2002)).

### 5.2. Estimation

In order not to induce restrictions on the mechanisms that may run counter to the actual causal structure, the estimation must satisfy two requirements. Firstly, it should focus on time local quantities; secondly, it should be non-parametric since parametric models may impose arbitrary restrictions. The first requirement is fulfilled by using local characteristics, i.e. transition rates or intensity processes in the time continuous case and changes over time (increments) in the time discrete case. By assuming a non-parametric linear statistical structure on the local characteristics, we can analyse the direct and indirect effects and combine them into total effects over various pathways.

Corresponding for example to the model in Fig. 7, and assuming the absence of the unmeasured confounder *U*(*t*), we can set up the following linear structural equations:


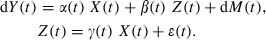
6

Here *M*(*t*) is a martingale. The difference between this and the usual structural equation models is that the first equation is defined for a differential (or a difference in the discrete case).

The estimation, which is called dynamic path analysis, takes place by successively regressing all variables on their parents in the graphical model. This analysis will be repeated for every event time when the outcome *Y*(*t*) is a counting process, or for every discrete time if *Y*(*t*) is a longitudinal data process. In the counting process case the analysis of the first regression, containing d*Y*(*t*), is done by using the additive hazard model, where the estimate for each jump time is added up to estimates for cumulative regression functions; see Aalen *et al.* (2008), chapter 9. The second equation, with *Z*(*t*) as dependent variable, is analysed by standard linear regression for each jump time of the counting process. For further details and analyses applied to real data, see also Fosen *et al.* (2006a, b) and Martinussen (2010). The analysis given in these references is analogous to that of ordinary path analysis based on time-dependent least square estimation. For longitudinal data the analysis follows the approach of Farewell (2006), Diggle *et al.* (2007) and Aalen and Gunnes (2010). In principle, the dynamic path diagram may be seen as continuously evolving over time, with edges possibly appearing or disappearing at any time when we collect new information. The validity of this approach will require similar assumptions to those of standard path analysis, but here extended to a time varying setting; for details, see Martinussen (2010), section 2.

### 5.3. Survival analysis and mediators–some further comments

In some fields the search for mechanisms through statistical analysis has been extensive, especially in psychology and social science where this activity received considerable inspiration from the highly quoted Baron and Kenny (1986). Emsley *et al.*(2010) redefined their approach in the modern causal inference setting, clarifying the necessary assumptions. In other areas mediation analysis has largely been ignored. This is especially so for situations where time plays a central role, as in survival analysis. In view of the importance of survival analysis in medicine and other areas, it is surprising that not more attention has gone into the issue of mediation.

The standard application of survival analysis is to use a Cox model with fixed baseline covariates. This implies that the data that are collected along the way, which could be very considerable in a clinical setting, are rarely incorporated in the survival analysis. Occasionally, time-dependent covariates are being used, but this creates difficulty if the covariates are internal, i.e. dependent on the underlying development in event risk, and if the main aim is to assess a treatment effect. In fact, the often massive information that is collected from patients in the time epoch between baseline and the occurrence of the event of interest is mostly ignored in the survival analysis. Clearly, this is a waste, especially since the data that are collected along the way (e.g. patient data) may contain information about how the treatment works. We argue that time-dependent covariates and markers should play a much larger role in survival analysis and that one should attempt to elucidate the relationship between these processes and the events. This actually corresponds to trying to understand the mechanisms of treatment; in which ways does treatment influence disease progression before the final event?

As an example, consider the homocysteine study of Bønaa *et al.* (2006). Homocysteine is an amino acid in the blood, and such that a high value is associated with a greater risk of heart disease. If this is really a causal factor, then we would expect that lowering homocysteine would decrease the risk. Bønaa *et al.* (2006) carried out a large randomized clinical trial, comprising 3749 men and women in four treatment groups. To decrease the homocysteine level, the vitamins folic acid (B_9_) and B_12_ were given, two groups receiving both of these vitamins: one together with vitamin B_6_ and one without B_6_. Two control groups were included, one placebo group and one receiving just vitamin B_6_. The surprising result was that, in spite of considerable lowering of homocysteine, the two groups receiving folic acid and vitamin B_12_ had no benefit with respect to heart disease and death; in fact those receiving also vitamin B_6_ had an increased risk of heart disease.

Clearly, when the results run counter to expectations there is a need to learn what happened and how the mechanistic understanding should be modified. In an editorial (Loscalzo, 2006) the following question was asked:

‘…if homocysteine is an atherogenic determinant, do the results of these trials suggest that vitamin therapy has other, potentially adverse effects that offset its homocysteine-lowering benefit?’.

This can be rephrased as asking whether there is an adverse ‘direct effect’ that offsets a possible beneficial indirect effect mediated through lowering of homocysteine. In fact, this is a question that could actually be analysed statistically when measurements of homocysteine during the study are available.

Hence, there is every reason to look more closely at the possible information in internal time-dependent covariates measured during survival studies, and dynamic path analysis is one possible tool.

### 5.4. Relationship between mediators and surrogate end points

One major medical application of the mediation concept is the idea of surrogate end points; see for example MacKinnon *et al.* (2007). When for example cholesterol lowering medication is evaluated, then the actual evaluation of ‘hard’ end points, such as myocardial infarction or death, is often not undertaken because it requires large and long-term studies. It is much simpler and faster to evaluate the effect of the medication on the level of cholesterol. The idea is that, if this level goes down, we might also expect an effect on hard end points, i.e. cholesterol is seen as a mediator of the effect of medication on the hard end points. Ideally, all effect should go through the mediator with no direct effect from the medication to the hard end points. This clearly requires a strong concept of indirect effect, such as the counterfactual effect that was discussed above; it will actually be true that when one intervenes on the cholesterol, say, then there is the expected effect on the hard end points.

## 6. Analysing data from the Swiss HIV Cohort Study

We shall illustrate some aspects of the theory by analysing data from the Swiss HIV Cohort Study. This is an on-going multicentre study following up HIV-infected adults aged 16 years or older (Ledergerber *et al.*, 1999). An important issue in this cohort is to study the effect of HAART which has been in use from 1996. Patients who died or refused further participation before 1996, who were on HAART or in clinical stage C at baseline, or whose treatment history before joining the cohort was uncertain are excluded from the analysis. The data are organized in monthly intervals, with measures of CD4 cell counts, HIV type 1 (HIV-1) ribonucleic acid (RNA) (viral load) and some other clinically relevant measures. For our analysis, once treatment is first initiated it is assumed that the individual remains on treatment from then on, as in Sterne *et al.* (2005) and Gran *et al.* (2010). Laboratory measurements are collected every 3 months on average. In total, 2161 individuals contributed to the data used in our analysis. The total observation time for one individual varied from 1 to 92 months. 202 of these individuals progressed to acquired immune deficiency syndrome or death, and 717 were treated with HAART.

CD4+ T-lymphocytes (CD4 cells) are part of the immune system and the main target of HIV-1 which makes them ideal markers for HIV disease progression. In contrast, HIV-1 RNA in plasma is an easily accessible marker for viral replication. HAART, defined as at least three drugs from at least two different classes, can reduce HIV-1 RNA levels in plasma by several orders of magnitude, often to values below the limit of detection of current assays. The treatment does not completely eliminate the virus from all body compartments but keeps viral replication at a very low level.

**Figure 9:**
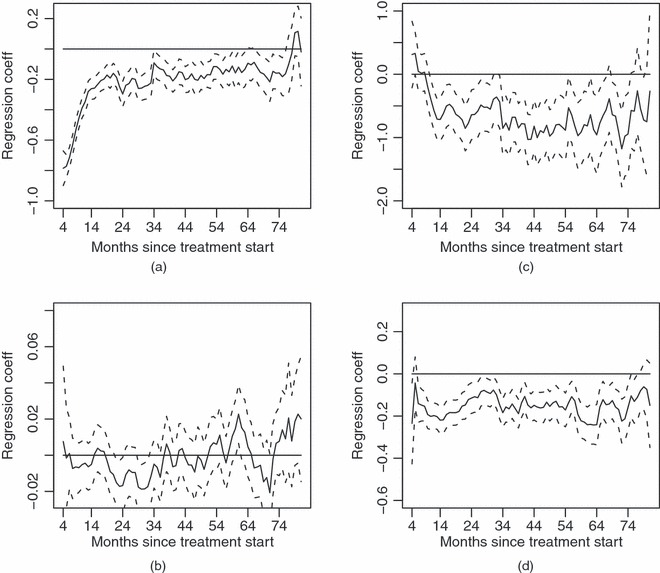
Patients on HAART: two regression analyses are presented with (a), (b) increments of HIV-1 RNA and (c), (d) increments of CD4 cell values as dependent variables, and lagged values of HIV-1 RNA and CD4 cell values as independent values; the analysis is performed for each month

**Figure 10:**
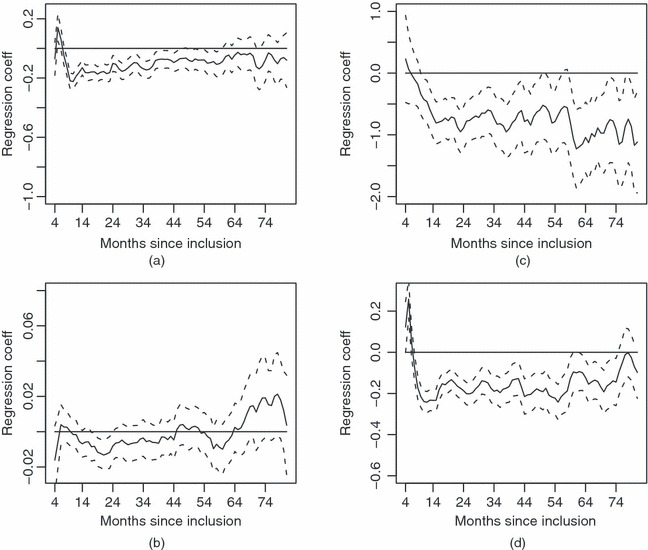
Patients not on HAART: two regression analyses are presented, with (a), (b) increments of HIV-1 RNA and (c), (d) increments of CD4 cell values as dependent variables, and lagged values of HIV-1 RNA and CD4 cell values as independent values; the analysis is performed for each month

As an illustration we shall analyse data on CD4 cell count and HIV-1 RNA for patients on treatment and patients not on treatment. In the first group we would expect that the primary process is the reduction of HIV-1 RNA, and that this then drives the increase in the CD4 cell count, i.e. we would expect that CD4 is locally dependent on HIV-1 RNA, but not the other way round. In Fig. 9 we present results of regression analyses performed at every month where dependent variables are increments of HIV-1 RNA and CD4 cell counts respectively, and where the independent variables are in both cases lagged values of HIV-1 RNA and CD4 cell counts. The analyses show (Fig. 9(c)) that lagged values of HIV-1 RNA have a significant effect on the CD4 cell count increments, whereas lagged values of CD4 do not have an effect on HIV-1 RNA increments (Fig. 9(b)). This indicates that the CD4 process is locally dependent on the HIV-1 RNA process, whereas in the opposite direction there is local independence. This fits with the biological understanding of how the treatment works: that it decreases the viral load which then drives an improvement of the immune system. Fig. 10 presents the same analysis for the untreated group and we see similar results. This is not surprising: although there is no treatment to lower the viral load, still the amount of virus may change over time and be a driving force for changes in the CD4 cell level.

The present figures show that simple analyses using a linear increment model (Diggle *et al.*, 2007) can give results that fit well with the mechanistic understanding of how changes in viral load influence the immune system. This gives reason to think that similar analyses could also contribute to mechanistic understanding in other fields. Clearly, the field of HIV infection is better understood than other chronic diseases, simply because of the major role of a known infectious agent. Most chronic diseases, such as cancer (in general) and heart disease have no such simple major cause, and there is even greater reason to attempt a dynamic statistical analysis of observable data.

## 7. Discussion

The idea of this paper has been to discuss in a broad way how the concepts of causal modelling and intervention can be viewed from a dynamic point of view. We believe that causality is intimately connected with time and that the process viewpoint is essential. We have pointed out that local independence and the associated graphs are relevant when viewing causal associations from a mechanistic point of view. The concept of local dependence and independence seems to capture in a very broad sense what happens when processes influence one another. Clearly, there is need for much more work in this area. Pathways are becoming of great importance in the field of systems biology, and statisticians ought to follow up and propose ways to understand and analyse such systems.

Although important and useful, the tendency in much causal modelling to focus only on marginal models, via elaborate weighting procedures, may have limitations. Specifically, these procedures do not bring out details about the underlying causal, or mechanistic, structure. We need to learn as much as is possible from the data.

Survival analysis is a field of special interest because it concerns the detailed follow-up over time of individuals. The present day situation, when in a major field like survival analysis much valuable information collected as time-dependent covariates is simply ignored, is very unfortunate.
